# The Use of the Hemostatic Net in Asian Population: Hyperpigmentation and the Duration Required for Hemostatic Net Marking to Disappear

**DOI:** 10.1007/s00266-024-04561-6

**Published:** 2024-12-02

**Authors:** Apinut Wongkietkachorn, Nuttapone Wongkietkachorn

**Affiliations:** Plastic and Reconstructive Surgery Unit, QPrime Surgical Center, Bangkok, Thailand

**Keywords:** Hemostatics, Hyperpigmentation, Retrospective studies, Hematoma, Humans

## Abstract

**Introduction:**

There are major concerns about using the hemostatic net in Asian populations due to potential hyperpigmentation and the extended duration required for the markings to disappear. This study is the first to describe its use in this demographic and aims to determine the occurrence of hyperpigmentation from the hemostatic net and the time required for the markings to fade.

**Methods:**

A retrospective review was conducted in patients who had a hemostatic net applied during facial surgery between July 2022 and April 2024 at Qprime Surgical Center in Bangkok, Thailand. The hemostatic net was applied using 5-0 nonabsorbable materials with needle sizes of 16 mm or 19 mm.

**Results:**

A total of 300 patients were included in this study. No persistent hyperpigmentation from the hemostatic net was observed. The disappearance rates of the hemostatic net markings were as follows: 33.3% at 2 weeks, 10.0% at 3 weeks, 46.7% at 4 weeks, 3.3% at 6 weeks, and 6.7% at 8 weeks. Overall, 90.0% of the hemostatic net markings subsided within 1 month.

**Conclusion:**

The use of the hemostatic net in the Asian population studied resulted in remarkable prevention of hematomas after facelifts and no persistent hyperpigmentation, with the majority of the markings disappearing within one month, thereby reinforcing the efficacy and safety of this technique.

**Level of Evidence IV:**

This journal requires that authors assign a level of evidence to each article. For a full description of these Evidence-Based Medicine ratings, please refer to the Table of Contents or the online Instructions to Authors  www.springer.com/00266.

## Introduction

The hemostatic net has become an effective tool for hemostasis and skin redraping in facelifts [1]. However, there are major concerns about using the hemostatic net in Asian populations due to the potential for hyperpigmentation [2]. Additionally, our patients have expressed concerns about the duration required for the hemostatic net markings to disappear. These questions have remained unanswered, as no study has addressed them to date. This study is the first to describe the use of the hemostatic net in an Asian population. It aims to determine the occurrence of hyperpigmentation from the hemostatic net and the duration required for the markings to fade.

## Methods

### Study Design and Participants

A retrospective review was conducted on patients who had a hemostatic net applied during facial surgery between July 2022 and April 2024. This study took place at Qprime Surgical Center in Bangkok, Thailand. Inclusion criterion was having undergone a gliding browlift, facelift, or necklift with hemostatic net application. All patients were Asians and were well-informed about the hemostatic net before the procedure. Exclusion criteria included previous facial operations, threadlifts, illegal filler injections, significant facial scars, inability to adhere to the post-operative protocol, and non-compliance with routine follow-up visits. The patients with previous facial operations, threadlifts, and illegal filler injections were excluded because the surgery on these conditions required the need to remove previous scar tissues and foreign substances that were additional to normal surgical circumstances and might alter the findings. Patients with significant facial scars were excluded because their skin was shown to be more prone to scarification. The patients with an inability to adhere to the post-operative protocol were excluded because some patients did not come to remove the hemostatic net within three days, which could result in prolonged hyperpigmentation. The non-compliance with routine follow-up visits was excluded because this study aimed to verify the timing of the hemostatic net mark disappear and the exact timing could not be verified. Demographic data were collected including body mass index, diabetes mellitus, hypertension, dyslipidemia, smoking, and alcohol use. Written informed consent was obtained from all subjects.

### Hemostatic Net Application

The hemostatic net was applied using 5-0 prolene (Ethicon, Inc, Somerville, NJ). The needle sizes were 16 mm or 19 mm, which allowed for more superficial tissue penetration compared to the original description by Auersvald and Auersvald, which used a 26-mm needle [1].

To avoid the frontal branch of the facial nerve, the Pitanguy line was drawn. Any hemostatic net applied within the 1.5-cm boundary of the Pitanguy line was placed with very shallow bites, without the full intention of closing the space in the subcutaneous layer. In this small part of the temporal area, the hemostatic net’s purpose was not for hemostasis but to help to be a small part of the skin redistribution during a browlift; moreover, a secondary hemostatic effect was also observed to be achieved. Beyond this area, the hemostatic net served as the primary tool for skin redistribution, browlifting, and hemostasis.

### Post-Operative Protocol

The surgical sites and the skin area with the hemostatic of all patients were covered with gauze and bandages for the first 48 hours without the need to open or clean. Typically, follow-ups were conducted three days after the operation to have the hemostatic net removed. After removal, patients could wash their face and apply cream or lotion with their preferred choice of products. One week post-operation, patients returned for suture removal. They were advised to completely avoid sunlight for two weeks and to limit sun exposure for up to two months. If they needed to go outdoors, they were instructed to wear protective clothing to shield their face from sunlight. To observe the nature of the hemostatic net marks disappear, all patients were not provided with any treatment for hyperpigmentation.

### Outcomes

The primary outcome was the incidence of persistent hyperpigmentation from the suturing marks. Secondary outcomes included the duration for the suturing marks to disappear, skin irregularity, and hematoma. Patients were followed by the author weekly until the hemostatic net markings completely disappeared and to determine if any skin irregularity subsided. Hematomas were categorized into four grades: grade 1 for no hematomas, grade 2 for microhematomas that were not clinically significant, grade 3 for minor hematomas best evacuated by manual expression or aspiration, and grade 4 for significant volume hematomas requiring surgical drainage [3].

## Results

**Fig. 1:**
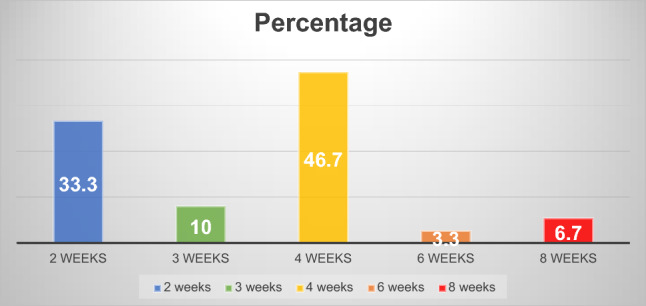
The duration required for the hemostatic net markings to disappear. It was found that 90.0% of the hemostatic net markings subsided within 1 month

There were 343 participants who met the inclusion criteria. There were 43 participants excluded, including eight previous facial operations, 12 threadlifts, three illegal filler injections, two significant facial scars, four inabilities to adhere to the post-operative protocol, and 14 non-compliance with routine follow-up visits. The final 300 participants were included in this study. The demographic data are shown in Table 1. No persistent hyperpigmentation of the hemostatic net markings was observed in this group. Figure 1 illustrates the duration required for the hemostatic net markings to disappear. We found that 33.3% disappeared at 2 weeks, 10.0% at 3 weeks, 46.7% at 4 weeks, 3.3% at 6 weeks, and 6.7% at 8 weeks.

**Table 1:** Demographic data (n=300)

Demographic data	N (Percent) or mean±SD
Age (years)	54.2±10.0
*Gender*	
Female	275 (91.7)
Male	25 (8.3)
BMI (kg/m^2^)	21.6±7.5
Diabetes mellitus	20 (6.7)
Hypertension	33 (11.0)
Dyslipidemia	40 (14.3)
Smoking	15 (5.0)
Alcohol use	10 (3.3)
*Procedure*	
Gliding browlift	243 (81.0)
Facelift	171 (57.0)
Necklift	96 (32.0)

**Fig. 2:**
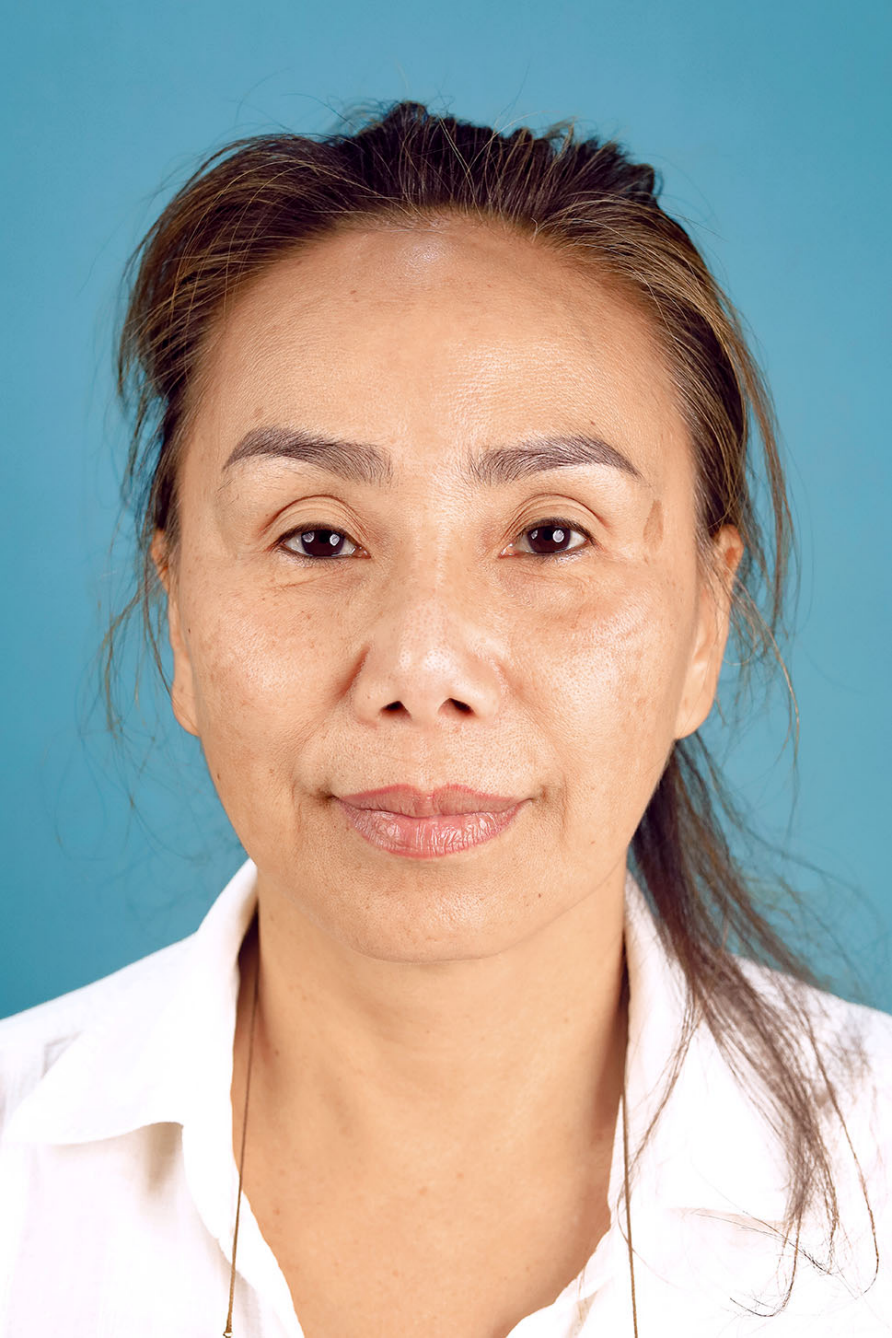
Preoperative photograph of a 63-year-old female undergoing a gliding browlift and facelift

**Fig. 3:**
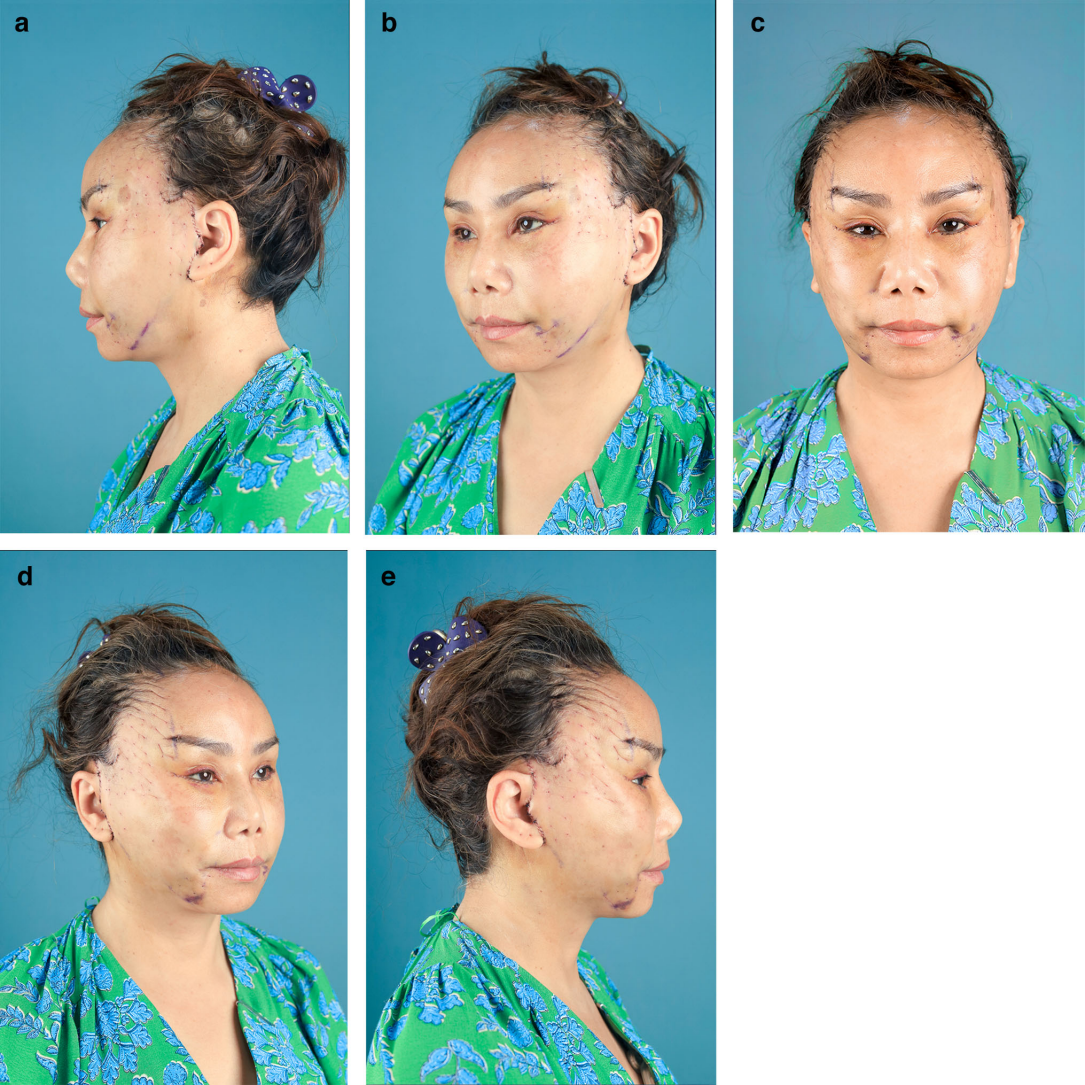
Anterior, oblique, and lateral views of the patient three days after the gliding browlift and facelift. The hemostatic net has been removed. **a** left lateral, **b** left oblique, **c** anterior view, **d** right oblique, **e** right lateral

**Fig. 4:**
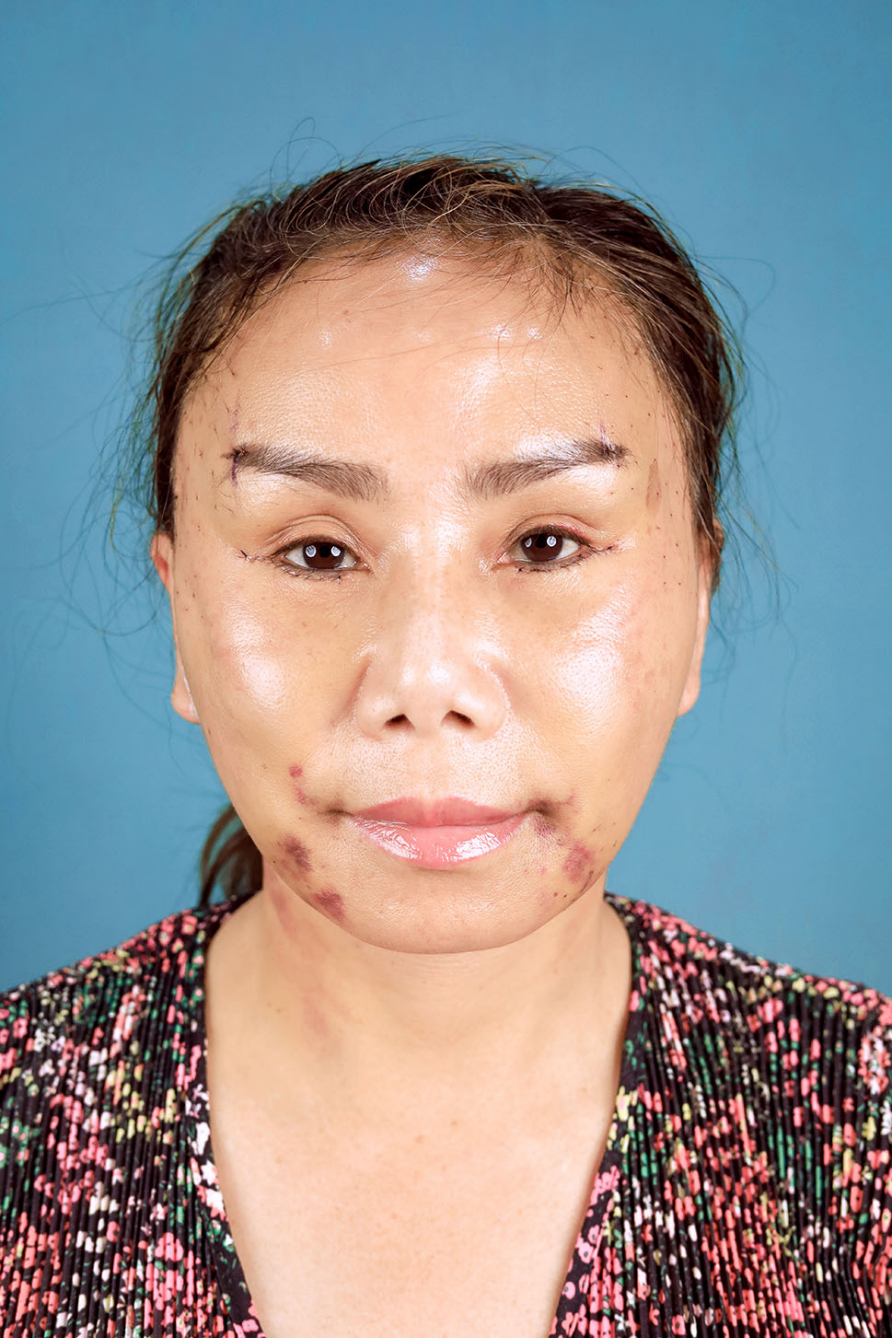
Seven days after the gliding browlift and facelift. The sutures have been removed

**Fig. 5:**
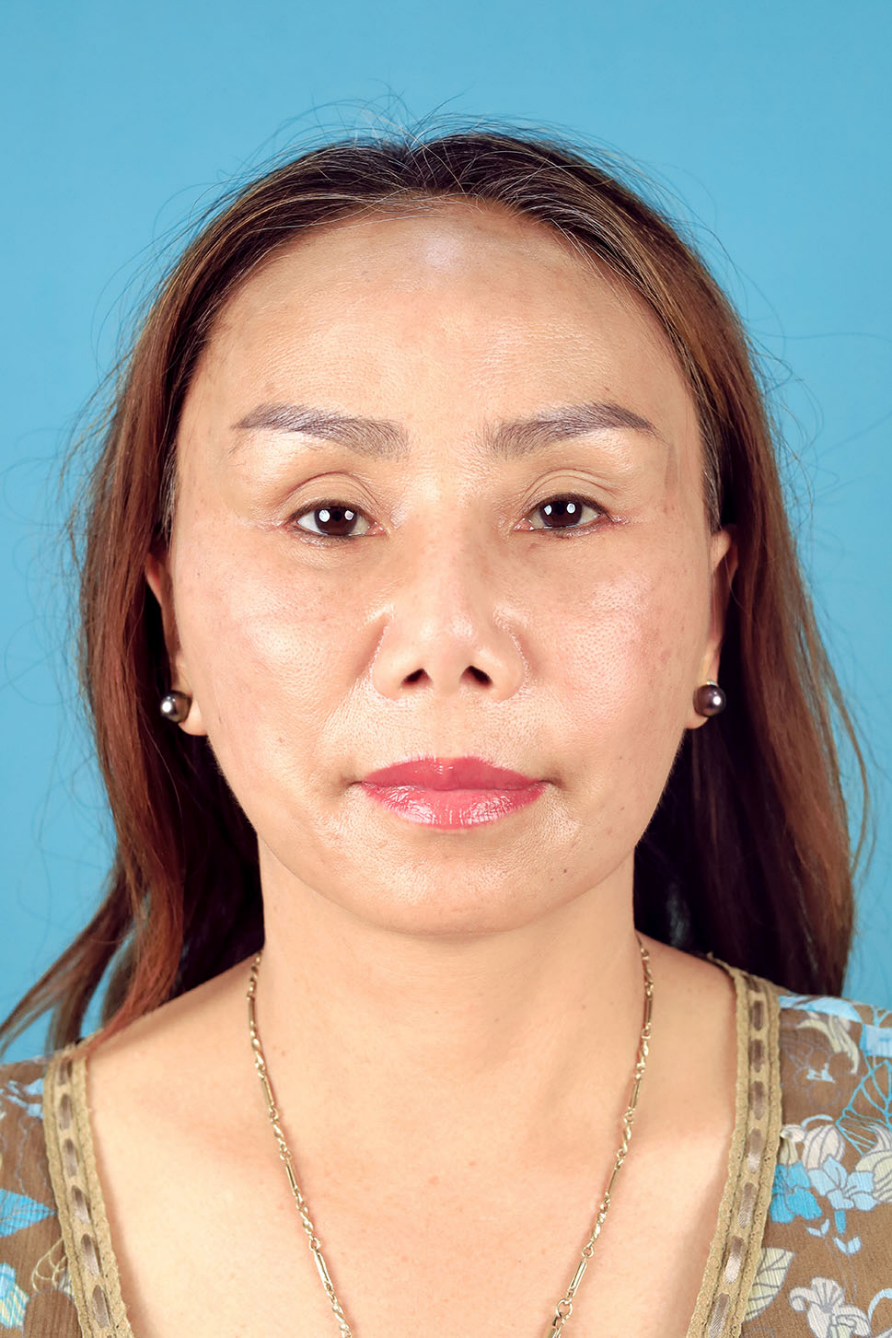
One month after the gliding browlift and facelift in a 63-year-old female

**Fig. 6:**
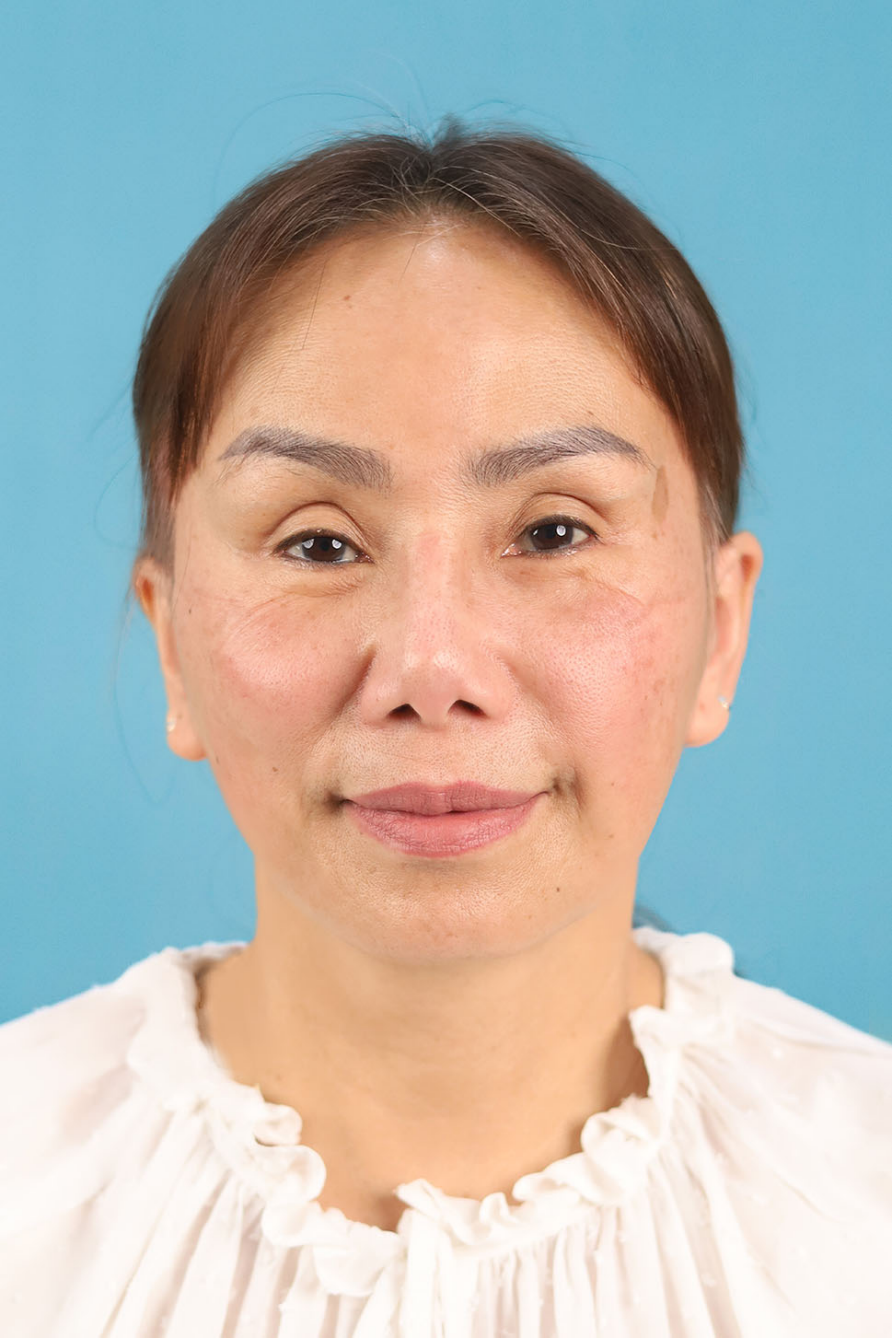
One year after the gliding browlift and facelift in a 63-year-old female

**Fig. 7:**
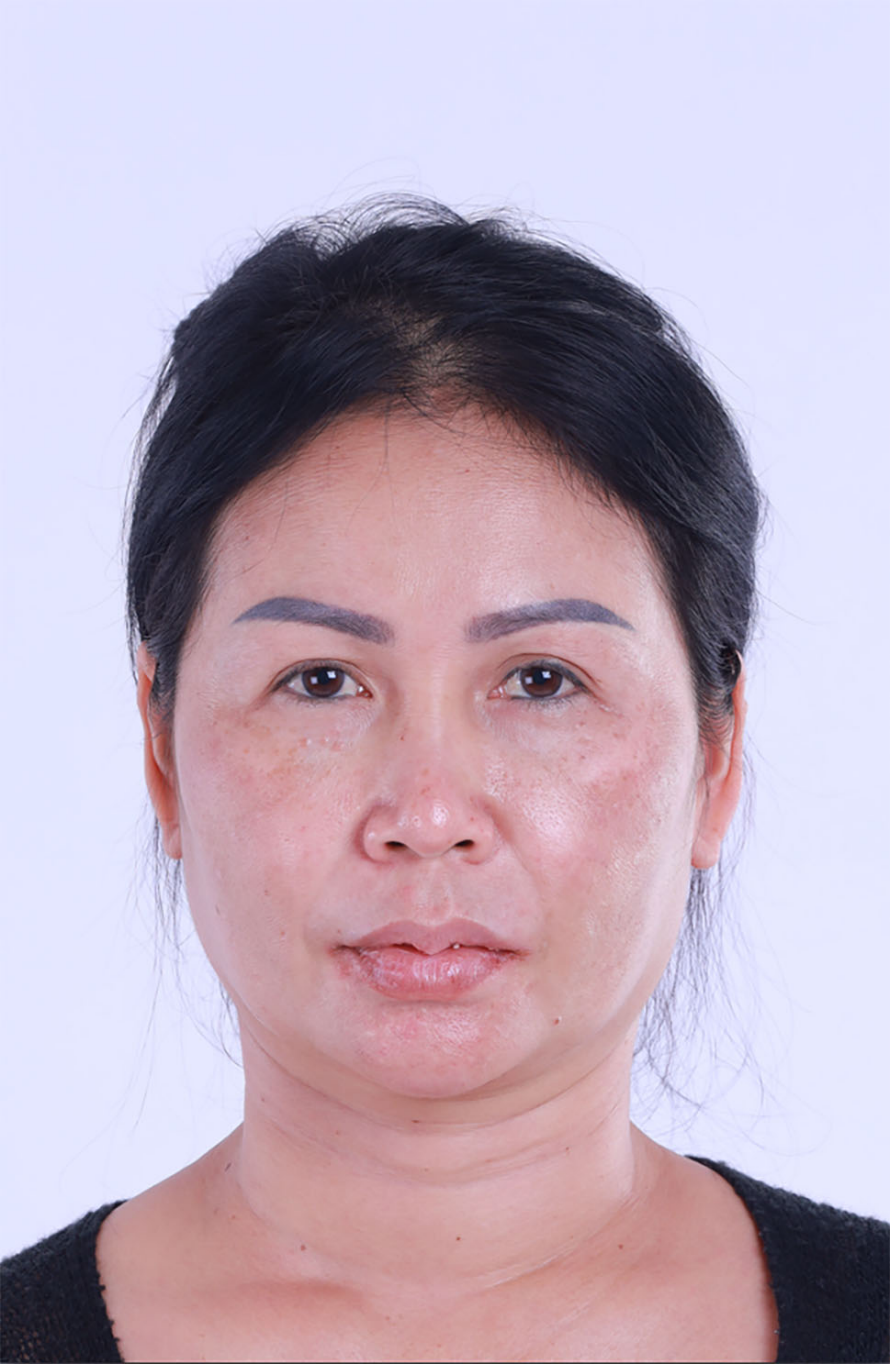
Preoperative photograph of a 42-year-old female undergoing a gliding browlift and facelift

**Fig. 8:**
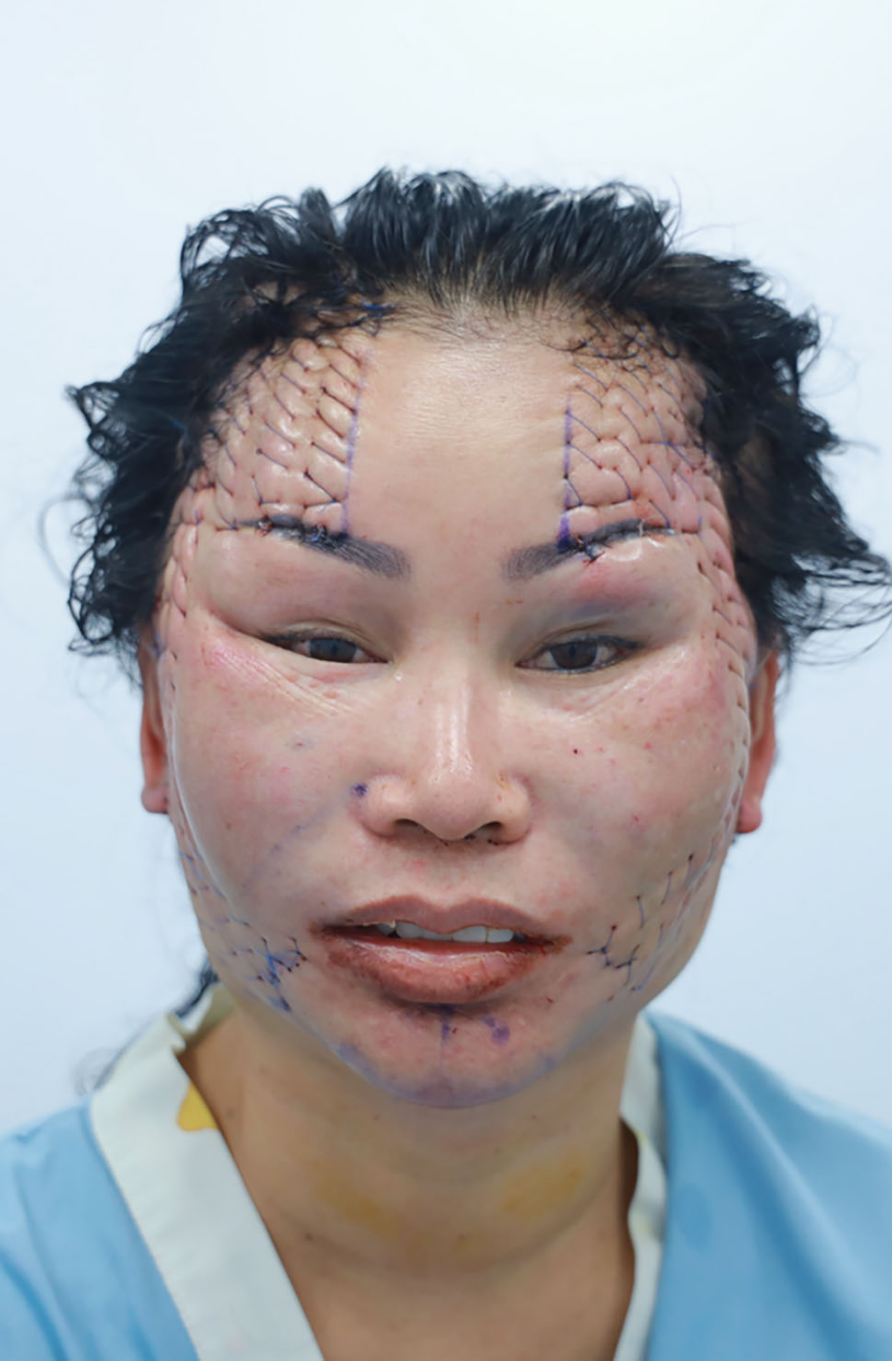
Immediate post-operative photograph of the gliding browlift and facelift. The hemostatic net was applied

**Fig. 9:**
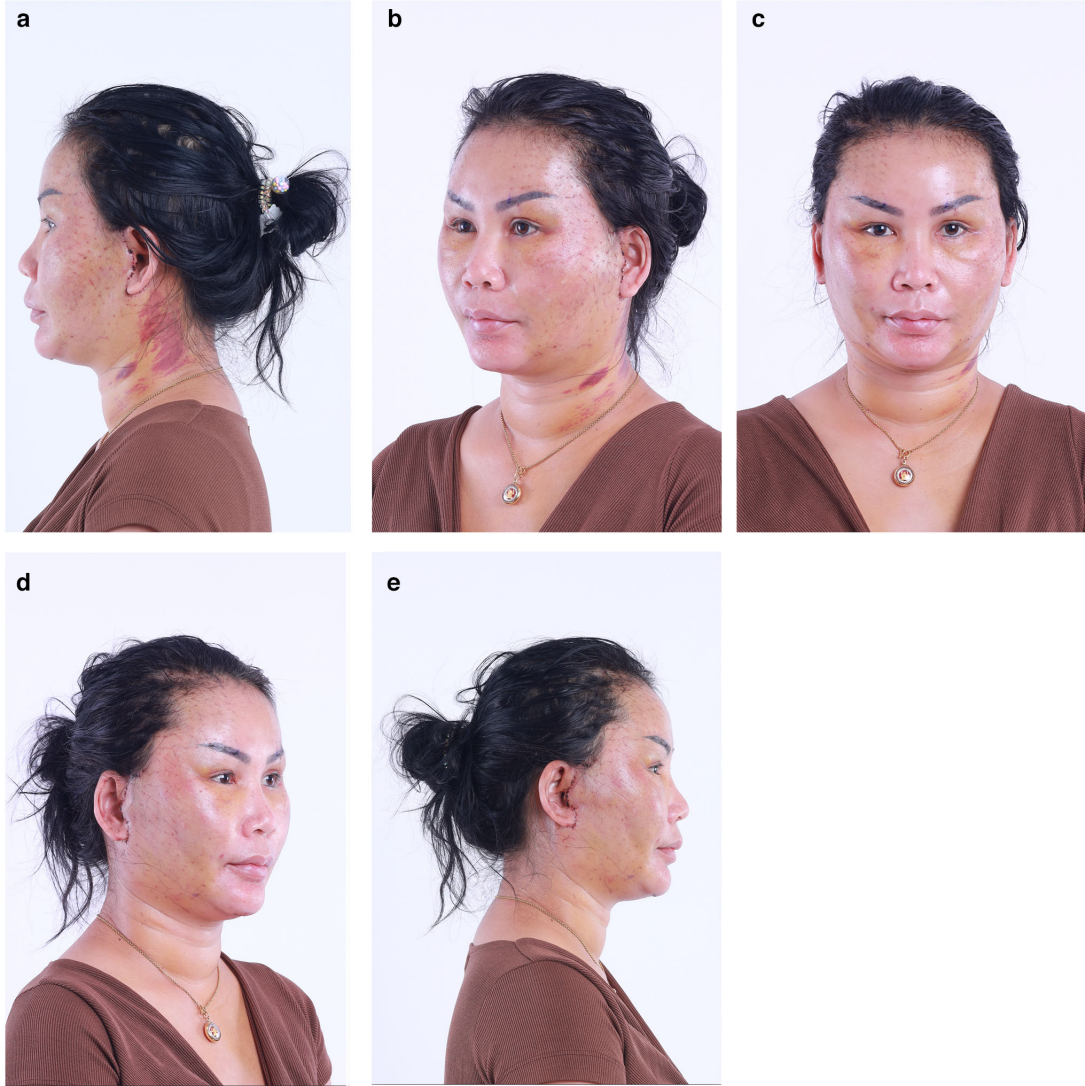
Anterior, oblique, and lateral views of the patient seven days after the gliding browlift and facelift. The sutures have been removed

**Fig. 10:**
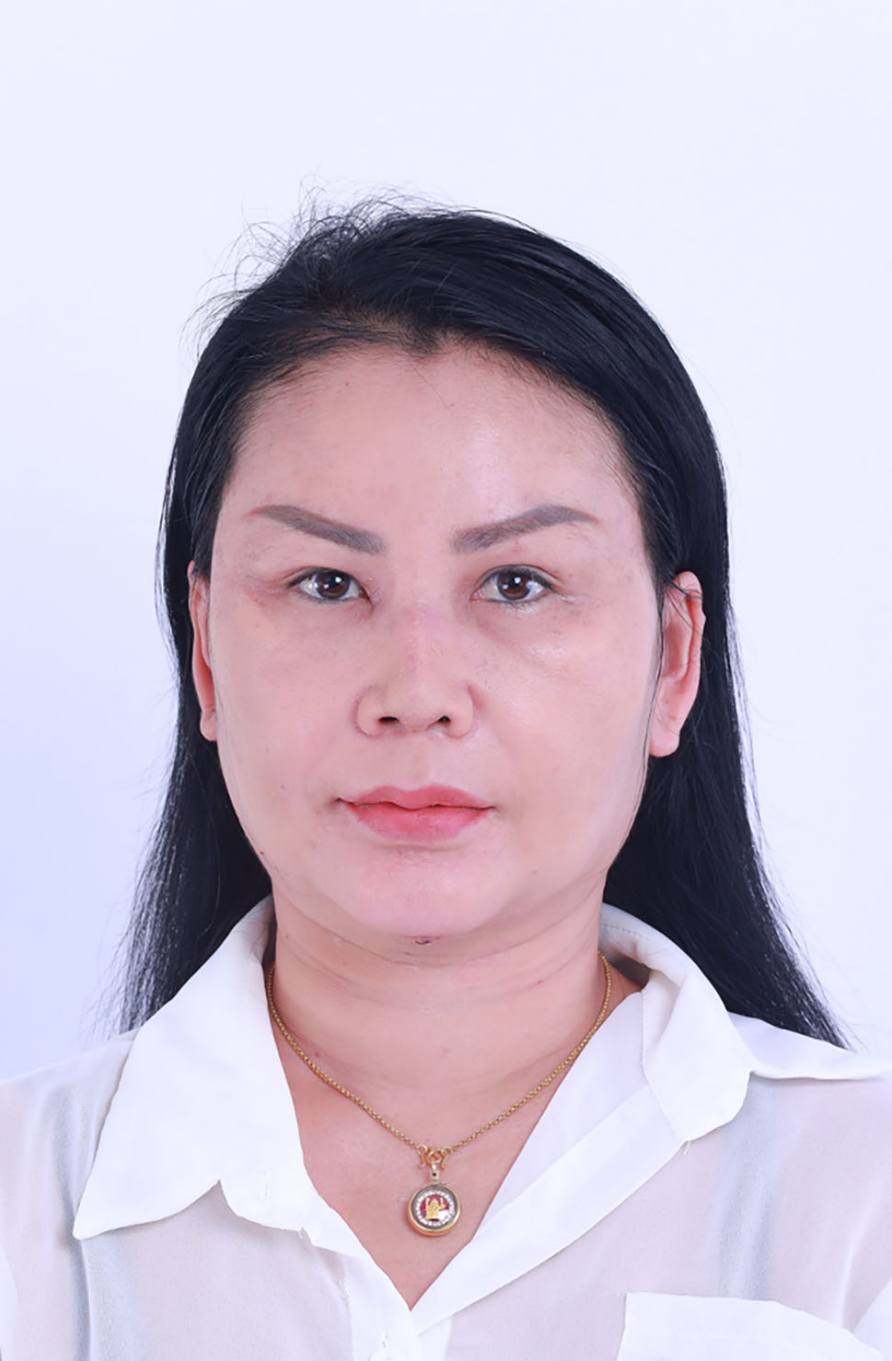
Six months after the gliding browlift and facelift in a 42-year-old female

In summary, 90.0% of the hemostatic net markings subsided within 1 month. There were two cases of skin irregularities that were self-resolving. Hematomas grade 3 were found in two cases and were resolved after aspiration. Other grades of hematomas were not found. Figures 2, 3, 4, 5, 6, 7, 8, 9, and 10 provide examples of the progression of hemostatic net markings and the outcomes of gliding browlifts in this Asian population. The different extent of hemostatic net application in the temporal region was to create different degrees and shapes of the browlift desired by the patients.

## Discussion

The main contributing factors to the results include the specific application method of the hemostatic net and the post-operative protocol. Only 5-0 sutures were used without any larger material, and the needle sizes were 16 mm or 19 mm, which are smaller than the original description [1]. This approach creates smaller holes and causes less trauma to the skin. The post-operative protocol of completely avoiding sunlight for 2 weeks and minimizing sunlight exposure for 2 months is also crucial. During the healing process, exposure to sunlight can increase the risk of hyperpigmentation [4]. Thus, avoiding sunlight is essential. Although this study was conducted in Thailand, which is just 15 degrees north of the equator and experiences significant sunlight, the longest duration of hyperpigmentation from the hemostatic net markings was still within 2 months. Using the hemostatic net in regions that receive less sunlight could potentially result in even faster recovery of the hemostatic net markings.

The use of the hemostatic net has been found to prevent hematomas, promote faster recovery, and help eliminate the need for drains [1]. This facilitates a quicker return to daily activities for patients and can serve as an effective promotional strategy for facelifts [1, 5]. Given that most hemostatic net markings were found to subside within 1 month, the hemostatic net, which enhances faster recovery, has the potential to become a powerful tool in future surgical procedures.

The main limitation of this study was that it included only patients who were thoroughly informed about the hemostatic net before surgery, ensuring they were aware of its use and the strict post-operative protocol, including avoiding sunlight. This adherence helped prevent issues during the study. However, if the hemostatic net is used on patients who are not fully informed or not prepared to follow the post-operative protocol, the results could differ.

## Conclusion

The use of the hemostatic net in the Asian population studied resulted in remarkable prevention of hematomas after facelifts and no persistent hyperpigmentation, with the majority of the markings disappearing within one month, thereby reinforcing the efficacy and safety of this technique.
